# Intrinsic Bipolar Head‐Direction Cells in the Medial Entorhinal Cortex

**DOI:** 10.1002/advs.202401216

**Published:** 2024-08-29

**Authors:** Xiaoyang Long, Xiaoxia Wang, Bin Deng, Rui Shen, Sheng‐Qing Lv, Sheng‐Jia Zhang

**Affiliations:** ^1^ Department of Neurosurgery Xinqiao Hospital Army Medical University Chongqing 400037 China; ^2^ Department of Basic Psychology School of Psychology Army Medical University Chongqing 400038 China

**Keywords:** bipolar head‐direction cell, medial entorhinal cortex, ring attractor network, unipolar head‐direction cell

## Abstract

Head‐direction (HD) cells are a fundamental component in the hippocampal‐entorhinal circuit for spatial navigation and help maintain an internal sense of direction to anchor the orientation in space. A classical HD cell robustly increases its firing rate when the head is oriented toward a specific direction, with each cell tuned to only one direction. Although unidirectional HD cells are reported broadly across multiple brain regions, computation modelling has predicted the existence of multiple equilibrium states of HD network, which has yet to be proven. In this study, a novel HD variant of bipolar HD cells in the medial entorhinal cortex (MEC) are identified that exhibit stable double‐peaked directional tuning properties. The bipolar patterns remain stable in the darkness and across environments of distinct geometric shapes. Moreover, bipolar HD cells co‐rotate coherently with unipolar HD cells to anchor the external visual cue. The discovery reveals a new spatial cell type of bipolar HD cells, whose unique activity patterns may comprise a potential building block for a sophisticated local neural circuit configuration for the internal representation of direction. These findings may contribute to the understanding of how the brain processes spatial information by shedding light on the role of bipolar HD cells in this process.

## Graphical abstract

Head‐direction (HD) cells function as the brain's internal compass. A classic HD cells fire preferentially when the head is oriented towards a specific direction. This study identifies a novel type of bipolar HD cells in the medial entorhinal cortex with intrinsically stable double‐peaked directional tuning. Their presence adds a new potential building block for spatial navigation.

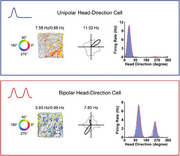

## 1 Introduction

Efficient navigation from one place to another requires our ability to maintain a sense of direction of the external environment, which is known to be presented by head‐direction (HD) cells in the brain.^[^ ^1^, ^2^ ^]^ An HD cell fires maximally and persistently when the animal's head is oriented toward a particular direction in a world‐centered reference system, and different HD cells exhibit different preferred directions.^[^ ^2^ ^]^ HD signals are generated in subcortical brain regions including the dorsal tegmental and lateral mammillary nuclei^[^ ^3^, ^4^, ^5^, ^6^ ^]^ and rely on intact vestibular input.^[^ ^3^, ^7^ ^]^ Then, the signals are widely transmitted to both subcortical and cortical areas including the anterodorsal thalamic nucleus, nucleus reuniens, postsubiculum, retrosplenial cortex, parasubiculum, medial entorhinal cortex and the sensory cortex.^[^ ^2^, ^8^, ^9^, ^10^, ^11^, ^12^, ^13^, ^14^, ^15^, ^16^ ^]^ The existence of cells responsive to the speed of angular head displacement (angular head velocity cells)^[^ ^10^, ^11^, ^17^ ^]^ and the connectivity patterns between the lateral mammillary nucleus and the dorsal tegmental nucleus^[^ ^18^, ^19^ ^]^ prompted the suggestion that a ring attractor network involving these structures may support the generation of HD signals throughout the brain, where attractor dynamics with local excitatory connections could sustain a localized activity bump.^[^ ^20^, ^21^, ^22^ ^]^

Previous theoretical work has examined the equilibrium states of the HD system in the ring attractor network model^[^ ^23^ ^]^ and revealed a diverse set of equilibrium states not only including the typical single‐peaked activity pattern, but also a double‐peaked activity pattern. Experimental studies have reported bidirectional HD tuning in the retrosplenial cortex and the parahippocampal region.^[^ ^24^, ^25^, ^26^ ^]^ A subset of HD cells in the retrosplenial cortex reversed their preferred firing directions when entering two visually distinct environments, thus exhibiting double‐peaked HD tuning.^[^ ^24^ ^]^ Investigation in the medial entorhinal cortex (MEC) and parasubiculum also revealed a distinct class of non‐rhythmic HD cells which showed alterations in the tuning curves when changing visual patterns were presented.^[^ ^26^ ^]^ Besides, a new type of axis‐tuned cells in the subiculum was reported and these cells fired consistently whenever the animal moved along a travel route associated with either one of the two preferred directions.^[^ ^25^ ^]^ However, the above activities were mainly attributed to the modulation of HD tuning by external environmental features rather than intrinsic double‐peaked activity, since these cells lost their directional tuning in an open field, unlike the classical HD cells. Until now, evidence supporting the existence of bipolar HD cells has been lacking.

The MEC lies at the top of the HD circuit organized in a hierarchy scheme,^[^ ^27^ ^]^ which implies a more sophisticated processing of HD signals. To investigate the possible existence of intrinsic bipolar HD signals in the MEC, we sought to explore whether HD cells could inherently exhibit bipolar tuning properties in a single visually stable environment. We found that MEC HD cells could mainly be divided into two functionally distinct classes: the classic single‐peaked HD cells and the novel double‐peaked HD cells, with occasional triple‐polar or quadruple‐polar HD cells. Remarkably, bipolar HD cells are expressed instantly when the animal enters an environment and are co‐active with classic HD cells. Intriguingly, the separation between the two peaks is clustered ≈90° and 180°, and remains stable despite the dynamic change of amplitude between the two peaks. The bipolar tuning properties remains unaltered in the darkness and across environments of distinct geometric shapes. Moreover, bipolar HD cells co‐rotate coherently with unipolar HD cells in response to the rotation of external visual cues. Despite their distinct tuning properties between the unipolar and bipolar HD cells, they show similar theta‐rhythmicity, which is in contrast to the previous functional classification of MEC HD cells based on their theta‐rhythmic activity.^[^ ^26^ ^]^ Altogether, these finely tuned bipolar HD cells in MEC confirm the existence of intrinsically stable double‐peaked HD activity and add to the diverse and sophisticated computational mechanisms of HD signals in MEC.

## 2 Results

### 2.1 Intrinsic Bipolar Head‐Direction Cells in the MEC

Neural activity was recorded from the MEC of six Long‐Evans male rats (Figure S1, Supporting Information) when they freely foraged for randomly scattered food pellets in the open arena. A total of 1282 putative single‐units were obtained, and HD cells were identified if both their mean vector length and angular stability exceeded the 99th percentile of the corresponding shuffled population distribution (Figure S2, Supporting Information). Angular stability was quantified by calculating the correlation coefficient between directional tuning curves from the first and second halves of the same recording trial. Using the above criteria, we reported that 383/1282 (29.88%) of the cells were classified as HD cells and the HD selectivity was stable within each recording session.

**Figure 1:**
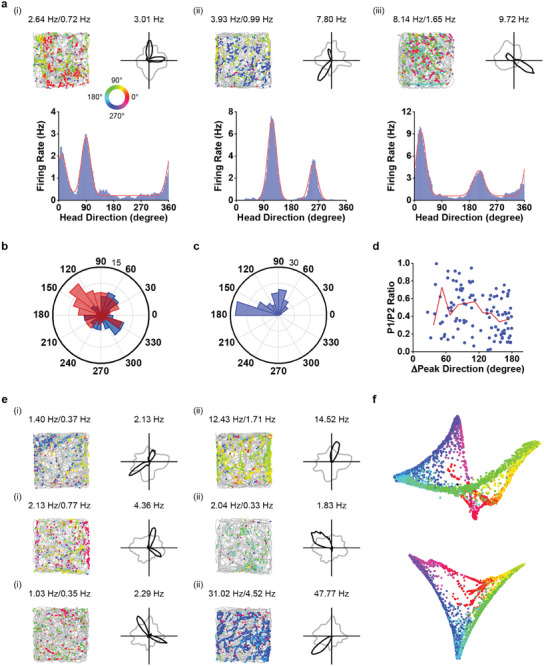
Bipolar head‐direction cells in MEC. a) Three bipolar HD cells from MEC. Left panel, color‐coded trajectory (grey line) with superimposed directional spike locations (Color circles indicate the corresponding head direction. Color bar shows the directional range of 0°–360°. Right panel, HD tuning curves (black) plotted against dwell‐time polar plot (grey). Peak firing rate, mean firing rate, and peak angular rate for each cell are labelled at the top of the panels. Bottom panel, histogram showing the directional distribution of firing rate across 360° with a bin width of 3° (purple) and an overlying curve showing a two‐component circular normal distribution fit (red). b) Polar plot showing the preferred directions for two peaks of all bipolar HD cells. Blue indicates the first peak with a higher angular firing rate. Red indicates the second peak with a lower angular firing rate. c) Distribution of angular offsets between the two peaks for all bipolar HD cells. d) Scatterplot showing the relationship between angular difference versus the ratio in angular firing rate between the two peaks. Red line showing the mean value binned at 15°. e) Three pairs of co‐recorded unipolar and bipolar cells. f) A neural manifold from the population activity of bipolar HD cells forms a topological ring. Upper panel, 3D structure. Bottom panel, two‐dimensional structure. Each dot corresponds to the neuronal activity at a single time. Color coding represents the parameterization of the manifold by head direction.

However, in addition to the classic single‐peaked HD cells, we also identified a novel type of bipolar HD cells. Due to the limited spatial resolution of the recovered electrode track, clear differentiation between layers IV, V, and VI was not always achievable. To simplify, we collectively refer to all deep layers IV/V/VI as deep layers. Previous studies have shown that unipolar head‐direction cells were mainly found in layers III to VI.^[^ ^13^, ^28^ ^]^ Concordantly, we found 370 out of 383 unipolar head‐direction cells were recorded from deep layers, whereas bipolar head‐direction cells (104 out of 106) were also prevalent in deep layers (Figure S3a, Supporting Information). Both percentages in deep layers were significantly higher than those in superficial layers II/III (Chi‐squared test, *P* < 0.001, Figure S3b, Supporting Information). To quantify the tuning properties of bipolar HD cells, we used a two‐component circular normal distribution^[^ ^21^ ^]^ to model the stereotyped triangular or Gaussian‐like shape of the HD tuning curves. Bipolar HD cells were identified if they met the following criteria: 1) the coefficient of determination (R^2^) of the fit exceeding the 99.9th percentile of the shuffled population distribution; 2) the ratio between peak firing rates of the small peak versus the large peak larger than 0.1; 3) the angular stability exceeding the 99th percentile of the shuffled population distribution. Using the above criteria, we reported that 106/1282 (8.27%) of the cells were classified as bipolar HD cells, and the proportions of bipolar HD cells were significantly larger than expected by chance (*Z* = 26.16, *P* < 0.001; binomial tests with expected *P*_0_ of 0.01). The bipolar directional tuning was not due to the recording artifact from two HD cells, as quantified by the Euclidean distance between the center of mass for the two separated unipolar firing peaks (Figure S4a–e, Supporting Information). Moreover, we quantified the unit isolation quality of both unipolar and bipolar HD cells by calculating the isolation distance.^[^ ^29^ ^]^ Unipolar and bipolar cells showed similar distribution of isolation distance, indicating waveform discrimination did not vary significantly between these two types of HD cells (Mann–Whitney U test, P = 0.06, Z = −1.90, Figure S4f,g, Supporting Information). The bipolar HD selectivity was stable within each recording session (Figure S2a, Supporting Information), with the bimodal distribution of HD tuning curves remaining similar between the first and second halves. The dual tuning curves were mostly asymmetric, with a dominant peak with a larger angular firing rate (**Figure** **1**a), although the relationship between the dominant and subordinate peaks underwent dynamic change across recording sessions (Figure S5, Supporting Information). Moreover, the bipolar directional tuning was not due to inhomogeneous sampling biases as confirmed by the maximum‐likelihood approach.^[^ ^12^, ^30^ ^]^ The bipolar spatial tuning remained unaltered after applying the correction algorithm and was similar across four quadrants (Figure S6, Supporting Information).

To investigate whether bipolar HD cells stabilize rapidly or change gradually after entering the environment, we split the recording sessions into eight epochs, and found that bipolar HD tuning was expressed instantly and was stable from the onset of the recording session (Figure S7, Supporting Information). The preferred firing directions for the two peaks were distributed across 360° (Figure 1b). Intriguingly, the difference between the preferred firing directions of two peaks clustered ≈90° and 180° (Figure 1c). To investigate whether the ratio between the amplitude of two peaks relates to their angular offset, we calculated the ratio of the angular peak firing rate between the primary and secondary peaks and their corresponding angular offset, and found there was slightly negative correlation (correlation coefficient *r* = −0.35, *P* = 0.002, Figure 1d). Notably, unipolar and bipolar HD cells were frequently co‐recorded (Figure 1e), indicating they intermingle with each other in the same local neural circuits. To uncover the internal structure of the bipolar HD ensemble, we used Laplacian Eigenmaps (LEM) for non‐linear dimensionality reduction as previously described.^[^ ^31^ ^]^ Interestingly, we found that the manifold from the neuronal population activity patterns of bipolar HD cells formed a ring topology (Figure 1e), similar to that of unipolar HD cells.^[^ ^32^, ^33^ ^]^ This result suggests that the internal structure of ensemble activity patterns of bipolar HD cells might also confine to the ring attractor, which in turn could parametrically encode the animal's instantaneous head direction.

### 2.2 Stable Tuning of Bipolar Head‐Direction Cells in Darkness

The ring attractor model accounts for the HD cells ability to integrate rotation signals and maintain head orientation in darkness.^[^ ^34^ ^]^ Head‐direction cells preserve their preferred firing directions in darkness, indicating that their firing patterns can be maintained by self‐motion.^[^ ^1^, ^35^ ^]^ To evaluate the influence of darkness on bipolar HD cells, we compared the effect of total darkness on their double‐peaked HD tuning. Altogether, 16 bipolar HD cells recorded in 13 recording sessions from four rats were tested under light, dark and light conditions, among which five unipolar HD cells and three grid cells were recorded.

**Figure 2:**
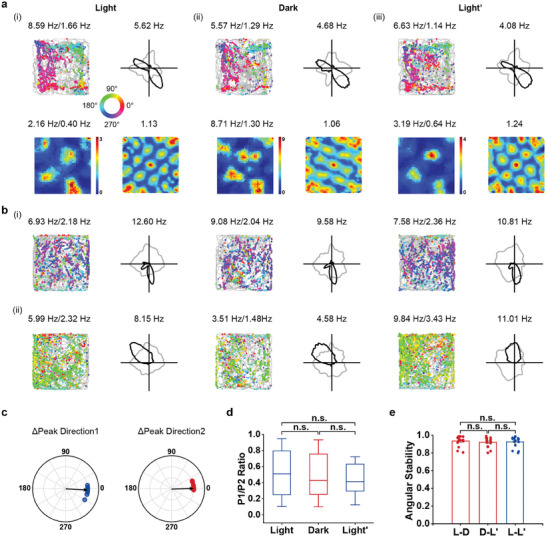
Persistence of bipolar head‐directional responses in the darkness. a) Co‐recorded one bipolar HD cell and one grid cell across three consecutive sessions (Light–Dark–Light). Notations and symbols are similar to Figure 1. b) Co‐recorded one bipolar HD cell and one unipolar HD cell. c) Polar plots showing the angular shift of two peaks of bipolar HD cells between light and dark sessions. Arrows indicate the median angular offsets. d) The ratio of peak firing rate between two peaks (P1 and P2) of bipolar HD cells remained stable across Light–Dark–Light conditions. e) Angular stability quantified by computing correlation coefficients of the HD tuning curves between light and dark sessions showing stable directional responses. All data are represented as mean ± s.e.m. unless stated otherwise.

Bipolar HD cells maintained their directional tuning in darkness, consistent with the stable spatial tuning of co‐recorded unipolar HD cell (**Figure** **2**b) or grid cell (Figure 2a). The preferred directions of two peaks of bipolar HD cells from light to darkness was similar (*n* = 16, peak1: median angular shift = −2.98°, circular V‐test for an angular shift of 0°, *V* = 15.78, *P* < 0.001; peak2: median angular shift = 1.49°, circular V‐test for an angular shift of 0°, *V* = 15.78, *P* < 0.001; Figure 2c), and the ratio of peak angular rate also remained stable (*n* = 16, Wilcoxon signed‐rank test, L‐D, *Z* = 0.31, *P* = 0.76; E‐B’, *Z* = 0.05, *P* = 0.96; L‐L’, *Z* = 0.26, *P* = 0.80; Figure 2d). Overall, the directionality of bipolar HD cells retained high angular stability (Wilcoxon signed‐rank test, L‐D versus D‐L’, *Z* = 0.68, *P* = 0.50; D‐L’ versus L‐L’, *Z* = 0.72, *P* = 0.47; L‐D versus L‐L’, *Z* = 0.68, *P* = 0.50; Figure 2e). This result is consistent with the previous study showing relative stable directional tuning of single‐peaked HD cells from MEC in darkness.^[^ ^36^ ^]^ Besides bipolar head‐direction cells, we found that co‐recorded grid cells tended to maintain their firing patterns in darkness (Figure 2a), consistent with the stable firing fields of grid cells in darkness in rats.^[^ ^37^, ^38^ ^]^ The maintenance of the grid firing patterns in the dark has led to the suggestion that grid cells could perform path integration based on self‐motion.^[^ ^39^, ^40^ ^]^ However, this result contrasts with degraded grid patterns under darkness in mice.^[^ ^36^ ^]^ The discrepancy might arise from the species‐specific difference between rats and mice exhibiting different dead‐reckoning capabilities.^[^ ^38^ ^]^ Taken together, MEC bipolar HD cells maintained their bidirectional pattern in darkness, supporting their ability to integrate self‐motion information in the absence of visual cues.

### 2.3 Sensory Control of Bipolar Head‐Direction Cells

**Figure 3:**
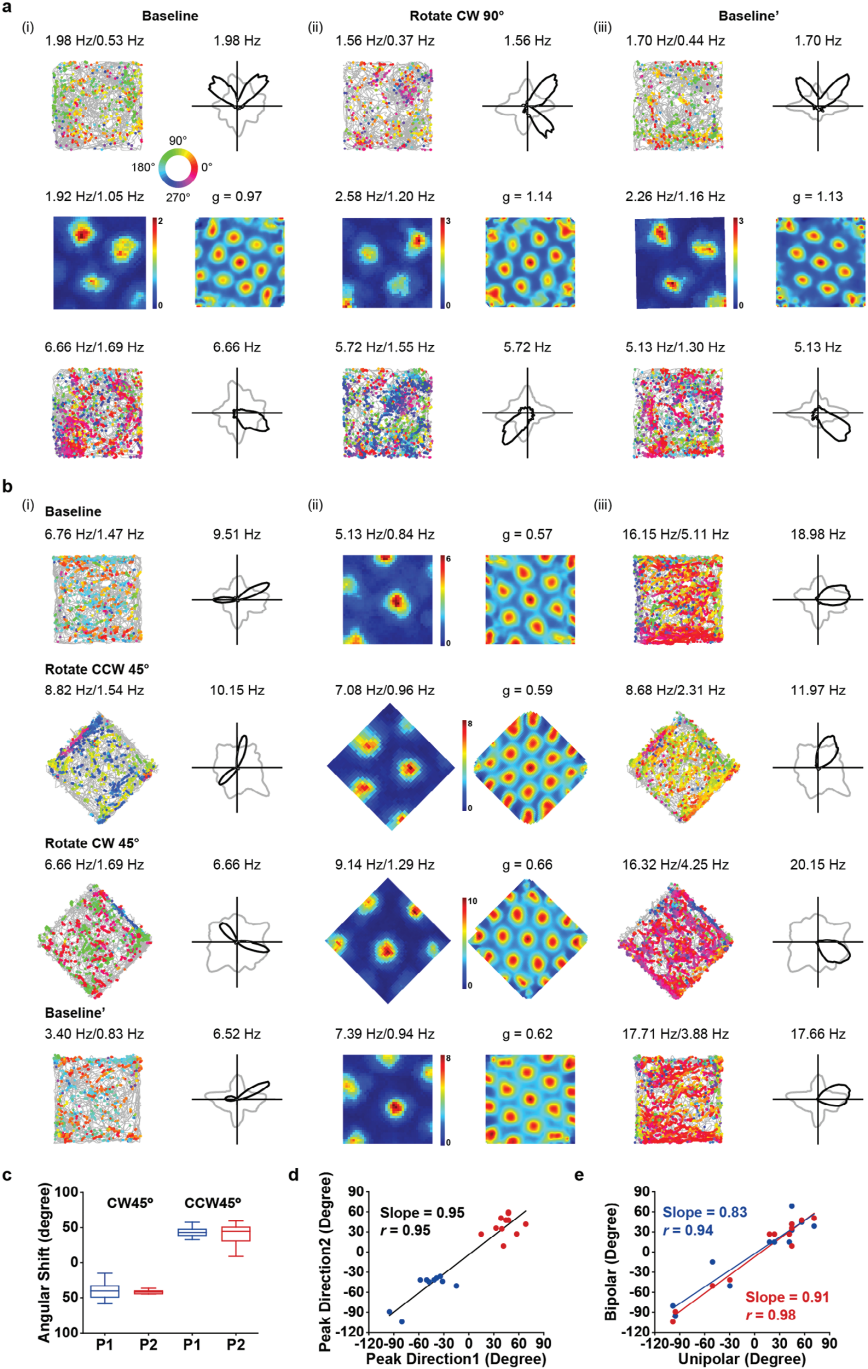
Responses of bipolar head‐direction cells to local cue. a) Spatial response of co‐recorded one bipolar HD cell, one grid cell and one unipolar HD cell to the 90° rotation of local cue. Notations and symbols are similar to Figure 1. For grid cells, firing rate maps and autocorrelation maps were shown. Firing rate was color‐coded with dark blue (red) indicating minimal (maximal) firing rate. The scale of the autocorrelation maps was twice that of the spatial firing rate maps. Gridness score (g) was labelled at the top of the panel. b) Spatial responses of co‐recorded one bipolar HD cell, one grid cell and one unipolar HD cell to the 45° clockwise or counter‐clockwise rotation of the whole arena. c) Angular shift of the preferred directions of two peaks (P1 and P2) of bipolar HD cells to the 45° clockwise (CW) or counter‐clockwise (CCW) rotation of local cue. d) Consistent shift of preferred directions for two directional peaks (Peak Direction 1 and Peak Direction 2) of bipolar HD cells. Linear fit was applied with the values for slope and correlation coefficient *r* indicated. Blue dots indicate CW rotation, while red dots indicate CCW rotation. e) Coherent shift of preferred directions for bipolar and unipolar HD cells. Blue and red dots represent data from two peaks of bipolar HD cells. Linear fit was applied with the values for slope and correlation coefficient *r* indicated.

Previous studies have shown HD cells shifted their preferred firing directions with the rotation of salient external cues.^[^ ^1^, ^35^, ^38^ ^]^ To test whether the bipolar directional tuning was dependent on local sensory cues, we recorded bipolar HD cells to the rotation of local cues. Co‐recorded bipolar HD cell, unipolar HD cell and grid cell rotated coherently to the 90° rotation of visual cue (**Figure** **3**a). When the whole arena was rotated either 45° clockwise or counter‐clockwise, co‐recorded bipolar HD cell, unipolar HD cell and grid cell also anchored coherently (Figure 3b), in line with a previous report.^[^ ^41^ ^]^ The preferred direction of two firing peaks of bipolar HD cells shifted consistently with the visual cue (Figure 3c) and the angular offsets between the two peaks remained unchanged (*n* = 13; correlation coefficient *r* = 0.95, Figure 3d). Moreover, directionality tuning of co‐recorded bipolar and unipolar (*n* = 9) HD cells drifted coherently (the first peak of bipolar versus unipolar, correlation coefficient *r* = 0.94; the second peak of bipolar versus unipolar, correlation coefficient *r* = 0.98; Figure 3e). This finding suggests a strong influence of local cues over unipolar HD cells as well as bipolar HD cells. The attractor network theory predicts coherent rotation of the preferred firing directions of classic HD cells,^[^ ^42^ ^]^ the above result further suggests the possible confinement of bipolar directional activity to the ring attractor dynamics.

### 2.4 Bipolar HD Cells are Insensitive to Environmental Geometry

**Figure 4:**
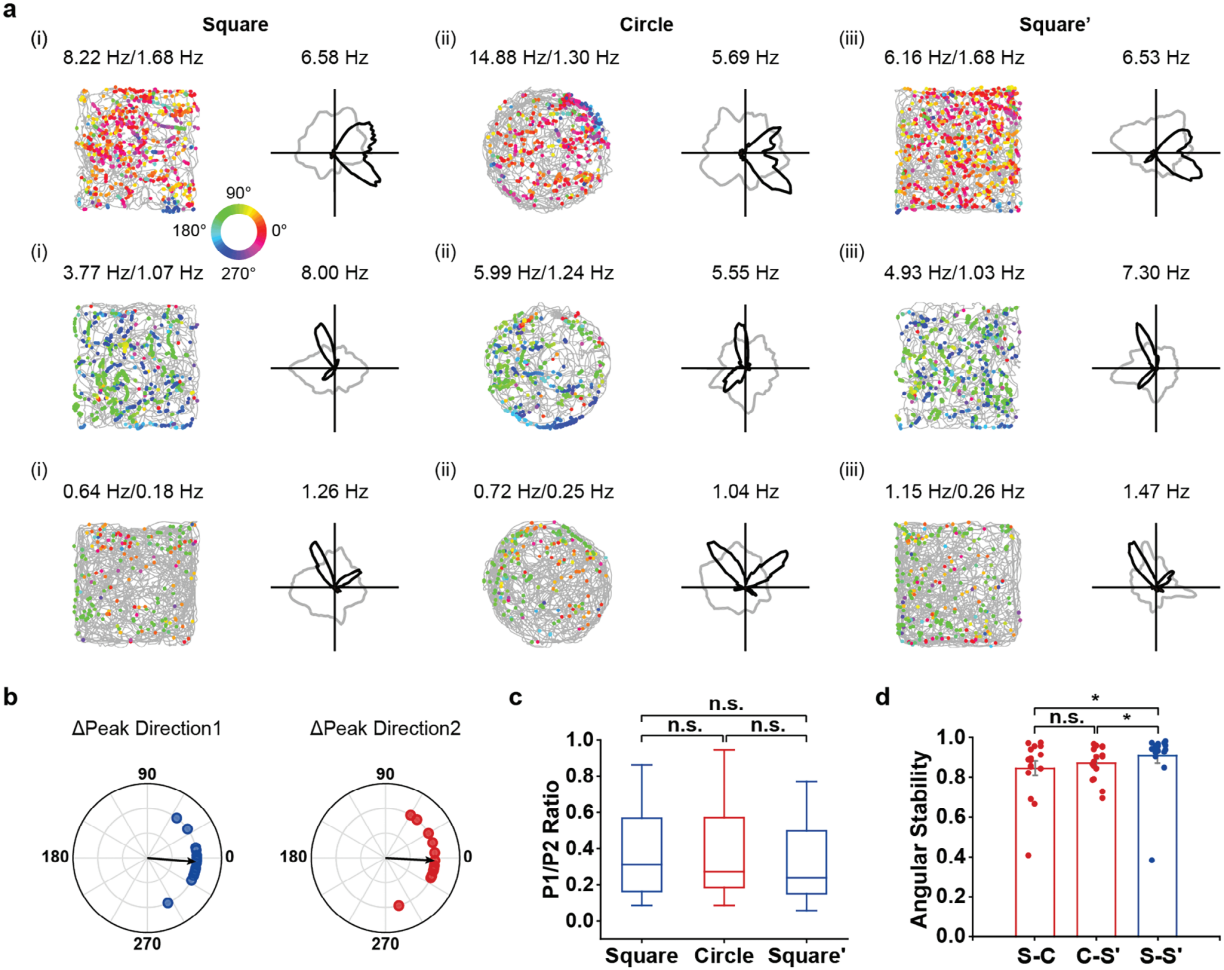
Responses of bipolar head‐direction cells in environments with different geometric shapes. a) Spatial response of three bipolar HD cells in square, circle and square enclosures. Notations and symbols are similar to Figure 1. b) Polar plots showing conserved preferred directions for two peaks of bipolar HD cells between square and circle enclosures. Arrows indicate the median angular offsets. c) The ratio between peak angular firing rates for two peaks of bipolar HD cells remained unchanged in environments with different geometric shapes. d) Angular stability across environments with different geometric shapes was stable.

A notable feature of bipolar HD cells was that the angular offset between two peaks is clustered ≈90° and 180°, which may reflect the unique environmental boundary features of the square enclosure. Previous studies have suggested the possible influence of geometry on classical unipolar HD cells,^[^ ^43^ ^]^ although the phenomenon was reported to be evident only in highly disoriented animals.^[^ ^44^ ^]^ As such, we next tested whether bipolar HD cells would maintain their preferred tuning in circular environments with uniform geometric boundary features (**Figure** **4**). We recorded 16 MEC bipolar HD cells as animals freely foraged in square, circular and square environments across three consecutive sessions (Figure 4a). The preferred direction of MEC bipolar HD cells were similar when the geometry of the environment was altered (*n* = 16, peak1: median angular shift = −4.46°, circular V‐test for an angular shift of 0°, *V* = 14.48, *P* < 0.001; peak2: median angular shift = −2.98°, circular V‐test for an angular shift of 0°, *V* = 13.94, *P* < 0.001; Figure 4b). Both peak ratio between two peaks of bipolar HD cells (*n* = 16, Wilcoxon signed‐rank test, S‐C, *Z* = 0.05, *P* = 0.96; C‐S’, *Z* = 1.19, *P* = 0.23; S‐S’, *Z* = 0.88, *P* = 0.38; Figure 4c) and their angular stability remained high to different shapes (Wilcoxon signed‐rank test, S‐C versus C‐S’, *Z* = 0.16, *P* = 0.88; C‐S’ versus S‐S’, *Z* = 2.28, *P* = 0.02; S‐C versus S‐S’, *Z* = 2.33, *P* = 0.02; Figure 4d). Thus, MEC bipolar HD cells are insensitive to environmental geometry.

### 2.5 Theta‐Rhythmicity of Bipolar HD Cells

Theta oscillations in the limbic system have been implicated to serve a critical role in the integration of spatial inputs.^[^ ^45^, ^46^, ^47^ ^]^ Theta rhythm and head‐directional firing accompany each other at various hierarchical levels along the pathway of HD signal propagation,^[^ ^12^, ^48^, ^49^ ^]^ and MEC lies at the top of the HD circuit.^[^ ^27^ ^]^ MEC neurons have been known to exhibit prominent rhythmic activity in the theta frequency band,^[^ ^12^, ^50^, ^51^ ^]^ and canonical HD cells fire in a theta cycle‐by‐cycle manner.^[^ ^52^, ^53^ ^]^ A previous study investigated how head‐directional firing is temporally modulated by theta rhythm and found that the highest degree of theta‐rhythmicity was evident when the animal was heading/facing in the preferred direction and theta rhythmic firing of HD cells correlated with their tuning properties.^[^ ^26^ ^]^

**Figure 5:**
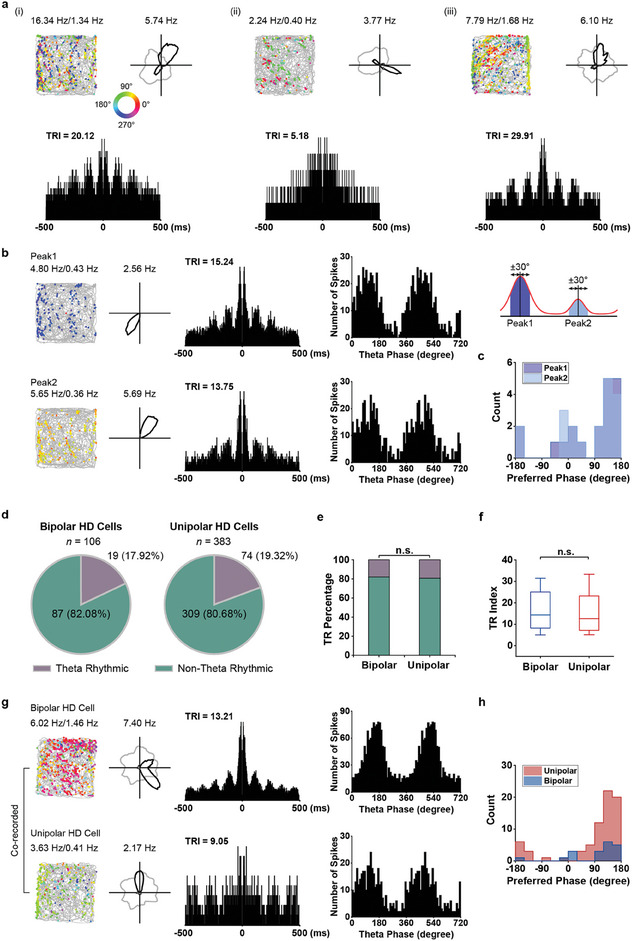
Theta rhythmicity of bipolar head‐direction cells in MEC. a) Three bipolar HD cells with theta‐rhythmicity. Top two panels, notations and symbols are similar to Figure 1. Bottom panel, spike‐time autocorrelograms of three bipolar HD cells. b) Theta rhythmicity for the two peaks of bipolar HD cells. The bipolar HD cell was split into two clusters based on two preferred directions, and theta rhythmicity and theta phase were computed separately for the dual peaks. c) Distribution of preferred theta phases for the two peaks of theta‐rhythmic bipolar HD cells. d) Proportion of bipolar and unipolar HD cells showing theta‐rhythmicity. e) The fraction of bipolar HD cells showing theta rhythmicity was similar to that of unipolar HD cells. Purple and green portions represent the proportions of theta‐rhythmic cells and non‐theta‐rhythmic cells, respectively. f) Comparison of theta rhythmic index (TRI) of theta‐rhythmic bipolar and unipolar HD cells (Mann–Whitney *U* test; Z = 0.43, *P* = 0.67). g) Theta rhythmicity and theta phase for co‐recorded one bipolar HD cell and one unipolar HD cell. h) Left, distribution of preferred theta phases for theta‐rhythmic unipolar and bipolar HD cells.

Here, we sought to characterize how theta rhythm influences the directional tuning of bipolar HD cells and whether theta rhythmic firing distinguishes the firing properties of unipolar HD cells from bipolar HD cells. We found that a subset of bipolar HD cells exhibited high theta power in their spike‐time auto‐correlogram spectra (i.e., theta rhythmic index; TRI, **Figure** **5**a) and was deemed to be theta‐rhythmic.^[^ ^54^ ^]^ Interestingly, the two peaks of bipolar HD cells exhibited similar theta‐rhythmicity and were locked to the same phase of theta cycle (Figure 5b,c). The proportion of bipolar HD cells that showed theta‐rhythmicity was similar to that of classic unipolar HD cells (17.92% vs 19.32%, Chi‐squared test; *P* = 0.75, Figure 5d–f). The extent of theta modulation has been reported to decrease from layer II to layer V, with 36/91 (39.56%) layer II neurons, 14/51(27.45%) layer III neurons and 16/105 (15.24%) layer V neurons exhibiting significant theta modulation.^[^ ^55^ ^]^ Another study reported a higher percentage of theta‐rhythmic HD cells in MEC.^[^ ^26^ ^]^ This discrepancy may be attributed to layer‐specific theta modulation, the majority of the latter recording sites were located in MEC layer II, while most cells in the current study were recorded from MEC deep layers. Since theta rhythm is known to coordinate MEC networks via theta phase relationships,^[^ ^53^, ^56^ ^]^ we next investigated the possible segregation of theta phase‐locking of unipolar and bipolar HD cells. By comparing their preferred firing phase, we found that at the population level, both types of HD cells tended to fire at the theta peak (Figure 5h). However, at the single cell level, co‐recorded unipolar and bipolar HD cells could exhibit phase differences of up to ≈50° (Figure 5g). Altogether, these results indicate bipolar HD cells exhibited coherent theta‐rhythmicity between the dual peaks, and might be differently entrained by theta phase as unipolar HD cells.

## 3 Discussion

In this study, we have identified a new population of bipolar HD cells within MEC. Unlike the classical MEC HD cells that exhibit single‐peaked Gaussian‐shaped directional tuning curves,^[^ ^13^ ^]^ bipolar HD cells show robust double‐peaked directional activities. Furthermore, this representation is stable under darkness, and is insensitive to the geometric shapes of the environment. Intriguingly, the separation between two peaks of bipolar HD cells is clustered ≈90° and 180°, which may reflect the intrinsic properties of the HD network. In addition, bipolar HD activities are coherently modulated with co‐recorded HD and grid cells by the external visual cue. Furthermore, bipolar HD cells showed similar theta‐rhythmicity as unipolar HD cells, with two peaks exhibiting the same theta phase. These properties of bipolar HD cells suggest that they could comprise as a potential building block in the spatial circuits of MEC and provide new insights into the neural representation of orientation in the mammalian brain.

By fully analyzing the equilibrium states of a generic ring attractor network, a widely used framework for the generation of HD signals, previous modelling work has predicted that the equilibrium states not only include the classic single‐peaked HD pattern but also double‐peaked HD pattern.^[^ ^23^ ^]^ In line with their prediction that the model can be generalized readily to generate activity patterns with three or more peaks, we also observed triple‐polar and quadruple‐polar HD cells (Figure S8, Supporting Information). Our study, for the first time, proves the existence of intrinsic bipolar HD cells. Additionally, Wang and Zhang suggested that a ring network with two activity bumps rotating at half of the speed as the single activity bump will still result in HD cells with a normal, single‐peaked tuning curve. However, if the rotation speed is the same, the ring network with two activity bumps should generate double‐peaked HD tuning curves (Figure S9, Supporting Information), and the separation between the two peaks would depend on the separation of the two activity bumps of the ring network. For instance, if the two activity bumps are 180° apart, then the bipolar HD cells would also exhibit two peaks that are 180° apart. However, these unique activity patterns would put specific requirements on structures of the two‐bump ring networks, which should have strong synaptic connections between cells with the separation between their preferred directions matching the separation of the two activity bumps. Motivated by this possibility, we performed cross‐correlograms for 96 co‐recorded HD cell pairs in 33 sessions across four rats. However, we found no obvious synaptic connections between unipolar and bipolar HD cells (Figure S10a–d, Supporting Information). On one hand, this lack of connectivity could arise from the angular disparities in preferred firing directions between unipolar and bipolar HD cells (median angle = 47.41°, Figure S10e, Supporting Information), since unipolar HD cells have been known to preferably connect with other HD cells with similar preferred directions.^[^ ^57^ ^]^ On the other hand, this may be due to the limitation of our recording techniques, which can only record a small number of HD cells at the same time. To further investigate the structures of the possible two‐bump ring networks, large‐scale monitoring of HD ensembles using high‐density silicon probe recording^[^ ^57^ ^]^ or grin‐lens‐based calcium imaging^[^ ^58^ ^]^ would be required. This will allow both anatomical connections and temporal correlation structures within HD circuitry to be revealed in great detail.

Previous studies have reported double‐peaked head‐directional activities.^[^ ^24^, ^25^, ^26^ ^]^ A group of neurons showed bidirectional firing patterns in a bidirectionally symmetrical environment in the dysgranular retrosplenial cortex,^[^ ^24^ ^]^ a brain region relaying environmental sensory information to the HD system.^[^ ^59^ ^]^ In their two compartments with opposing visual cues, a group of neurons called between‐compartment cells fired in one direction in one compartment, and in the opposite direction in the other compartment, following the cues. Another subset of neurons called within‐compartment cells fired in both directions in each compartment, regardless of the cues. Both types of bidirectional cells lost their directional tuning in an open field, suggesting that they depend on environmental landmarks. Another study reveals a new population of HD cells in the parahippocampal region including MEC and parasubiculum, showing bidirectional tuning with two preferred directions to two visual landmark manipulations.^[^ ^26^ ^]^ These bidirectional HD cells were non‐theta‐rhythmic, and showed non‐coherent changes as co‐recorded theta‐rhythmic HD cells. Notably, the two peaks were not always separated at a 180° angle to each other and the firing rate ratio of the two largest peaks in the tuning curve varied on a trial‐by‐trial basis, similar to the bipolar HD properties observed in the current study. In another study, a new type of axis‐tuned cells in the subiculum was reported and these cells fired consistently whenever the animal moved along a travel route associated with either one of the two preferred directions.^[^ ^25^ ^]^ Interestingly, the two different directions were always 180° apart from each other, and remained stably anchored to the global reference frame despite the rotation of the recording apparatus. Like the bidirectional HD cells, axis‐tuned firing was not observed in the open arena. In all, these bidirectional cells are thought to reflect a two‐way interaction between local and global direction signals, and to assess the stability of environmental landmarks, thereby determining their utility as reference points for the HD system. Altogether, these findings suggest that the HD signal in cortical areas is more heterogeneous than previously thought.

However, the bipolar HD cells identified in the current study differed from previously discovered bidirectional HD cells in several ways. First, the bidirectional HD cells were dependent on two distinct external cues, and they lost their bidirectional patterns in an open field with a single cue,^[^ ^24^, ^25^ ^]^ which is the primary setup we used to identify bipolar HD cells. Second, in visual cue manipulations, we found bipolar HD cells rotate coherently with simultaneously recorded classic unipolar HD cells, and differed from previously identified bidirectional HD cells which were independently modulated from classic HD cells.^[^ ^26^ ^]^ Third, unipolar and bipolar HD cells showed similar theta‐rhythmicity, unlike the non‐theta‐rhythmic bidirectional HD cells.^[^ ^26^ ^]^ Lastly, the separation between the primary and secondary peaks of the bipolar HD cells is not confined to a 180° angle to each other but rather distributed across 180°, in contrast to the fixed ≈180° angular offset of bidirectional axis‐tuned cells.^[^ ^25^ ^]^ Thus, bipolar HD cells reflect an intrinsic property of the MEC spatial circuit and add to the heterogeneous tuning properties of the head‐directional coding scheme.

Intriguingly, the orientation separation between the dual peaks of bipolar HD cells clustered ≈90° and 180°, with the number of cells being 180° apart outnumbering those of 90° by almost two times. Since the environmental boundaries of the square enclosure are orientated along two axes of the allocentric directions and forming 90° corners, we questioned whether this phenomenon was merely the influence of the geometric boundary. However, we found that bipolar HD cells maintained their preferred tunings and angular offsets in circular environments with uniform geometric features. Thus, the angular separation reflects an intrinsic property of the HD circuitry, which may be the result of specific synaptic connections between HD cells with preferred firing directions of 90° or 180° apart. Previous studies reported HD cells in anterodorsal thalamic nucleus (ADn), post‐subiculum (PoS) and MEC with adjacent preferred direction exhibited positive temporal correlations, whereas cells with opposite preferred direction were anti‐correlated and never fire simultaneously.^[^ ^57^, ^60^ ^]^ This result would not support the above‐mentioned structure of HD circuitry required for the generation of bipolar HD patterns. Therefore, further detailed investigation into the connectivity pattern of HD circuitry would be required.

What could be the potential functional significance of bipolar HD cells besides the classic unipolar HD cells? Bipolar HD cells may serve as a reliable reference for the animal's orientation, and encode multiple orientations at the single cell level through rate coding via the dynamic weight between two bump activities. Bipolar HD cells may also integrate task‐ or context‐relevant spatial information from different sources, by receiving inputs from distinct unipolar HD cells with their preferred directions matching the corresponding orientations of the two activity bumps of bipolar HD cells. Recent study has suggested HD cells in the anterior thalamic nuclei play a new role in relaying sensory and behavioral‐state information.^[^ ^61^ ^]^ Further investigations will be needed to elucidate whether bipolar HD cells are also involved in a broader cognitive process besides HD coding. Taken together, our findings reveal a new type of directional encoding scheme by bipolar HD cells, whose directional tuning plots are characterized by two distinct peaks in firing rate. Bipolar HD cells may constitute a new building block for spatial navigation.

## 4 Experimental Section

### Subjects

Six male Long‐Evans rats (2–4 months old, ≈250–450 g) were used for this study. Part of the data shares the same source of recorded animals (#MEC1‐#MEC5 in Figure S1, Supporting Information) of an unpublished paper of our own, which focuses on a distinct coding mechanism of allocentric and egocentric spatial coordinates in MEC (*Long* et al., *2024*, unpublished). All animals were housed in groups of four before surgery and singly housed in transparent cages (35 cm × 45 cm × 45 cm, W × L × H) and maintained on a 12‐h reversed light–dark cycle (lights on at 9 p.m. and off at 9 a.m.) after surgery. Experiments were performed during the dark phase. Rats were maintained in a vivarium with controlled temperature (19–22 °C) and humidity (55–65%). and were kept at ≈85–90% of free‐feeding body weight. Food restriction was imposed 8–24 h before each training and recording trial. Water was available ad libitum. All animal experiments were performed in accordance with the National Animal Welfare Act of China under a protocol approved with the permission license number #SYXK‐2017002 by the Animal Care and Use Committees from both Army Medical University and Xinqiao Hospital.

### Surgery and Tetrode Placement

Rats were anesthetized with isoflurane (2–3% mixed with oxygen), then fixed in a stereotaxic frame (David Kopf Instruments, Tujunga, California, USA) and kept on a heating pad to maintain body temperature at 37 °C. The local anaesthetic lidocaine was applied to the scalp before the incision was made. Microdrives loaded with four tetrodes were implanted to target the medial entorhinal cortex (MEC), 0.2–0.8 mm anterior to the transverse sinus, 4.5–4.7 mm lateral to the midline and 1.5–1.8 mm below the dura. Microdrive was slightly tilted in the sagittal plane at an angle of 10–12° in the anterior direction, and was secured with dental cement with 8–10 anchor screws, with one screw positioned behind the eye as a ground electrode. Each microdrive was composed of four tetrodes, which were assembled with four 17 µm Platinum/Iridium wires (#100167, California Fine Wire Company). The impedances of each electrode were reduced between 150 and 300 kΩ at 1 kHz through electroplating (nanoZ; White Matter LLC, Seattle, Washington, USA).

### Training and Data Collection

Behavioral training, tetrode advancement, and data recording started a week after surgery. Rats were trained to forage in a 1 m × 1 m square box with a white cue card (297 mm × 210 mm) mounted on one side of the wall, at a location midway between two corners with the bottom aligned to the floor. Food pellets were scattered into the arena intermittently to encourage exploration.

Each recording session lasted between 15 and 30 min to facilitate full coverage of the testing arena. Tetrodes were advanced in steps of 25 or 50 µm daily until well‐separated single units could be identified. Data were acquired by an Axona system (Axona Ltd., St. Albans, U.K.) at 48 kHz, band‐passed between.8–6.7 kHz and a gain of ×5–18k. Spikes were digitized with 50 8‐bit sample windows. Local field potentials were recorded from one of the electrodes with a low‐pass filter (500 Hz).

### Dark, Cue Rotation and Geometric Shape Sessions

Darkness sessions were used to evaluate the effect of the visual landmark on an HD cell's preferred firing direction. In the darkness experiment, the light was turned off, and the cue card was removed. The background luminance was reduced from 15 lux to nearly zero.

Another set of manipulation experiments was performed to evaluate whether bipolar HD cells can maintain their preferred firing directions across different geometric shapes including one 1 m × 1 m square box and one circular arena of 0.9 m diameter.

For cue rotation, MEC unit activities were first recorded in the standard recording session followed by a 45° rotation of the running box in the clockwise or counterclockwise direction. Next, another standard session was performed with the box rotated back to the original position.

The rats were kept in a holding enclosure between sessions. To remove any possible olfactory cues, the running environment was cleaned with an alcohol solution and water before each session. To reduce the possible influences of the surrounding environment, the rats were first disorientated and then placed them on the running box floor in a random direction.

### Spike Sorting, Cell Classification and Rate Map

Spike sorting was manually performed offline with TINT (Axona Ltd, St. Albans, U.K.), and the clustering was primarily based on features of the spike waveform (peak‐to‐trough amplitude and spike width), together with additional autocorrelations and cross‐correlations.^[^ ^62^, ^63^ ^]^ During our manual cluster cutting, neurons were always counted with similar or identical waveform shapes only once across consecutive recording sessions. To eliminate the possible artifact that spikes of bipolar HD cells belong to two individual unipolar head‐direction cells, it extracted two subclusters of each bipolar HD cell containing spikes emitted in the ±30° range surrounding each tuning curve peak and computed the Euclidean distance between their centers of mass in the scatterplot cluster‐space. The quality of unit‐isolation was quantified by calculating distances between clusters of different units in Mahalonobis space.^[^ ^29^ ^]^ Isolation distance was computed as the smallest ellipsoid from the center of the specific cluster which contained an equal number of noise spikes and cluster spikes. Thus, isolation distance measures the distance from a specific cluster to other clusters recorded on the same tetrode.

Two small light‐emitting diodes (LEDs) were mounted on the headstage to track the rats position and head orientation via an overhead video camera with the acquisition frame rate being 50 Hz. Only spikes with instantaneous running speeds > 2.5 cm s^−1^ were chosen for further analysis to exclude confounding behaviors such as immobility, grooming, and rearing.^[^ ^64^ ^]^

To classify firing fields and firing rate distributions, the position data was divided into 2.5‐cm × 2.5‐cm bins, and the path was smoothed with a 21‐sample boxcar window filter (400 ms; ten samples on each side).^[^ ^48^, ^64^ ^]^ Cells with > 100 spikes per session and with a coverage of >80% were included for further analyses. Maps for spike numbers and spike times were smoothed with a quasi‐Gaussian kernel over the neighboring 5 bins × 5 bins. Spatial firing rates were calculated by dividing the smoothed map of spike numbers by spike times. The peak firing rate was defined as the highest rate in the corresponding bin in the spatial firing rate map. The mean firing rate was calculated from data collected over the whole session. The spatial autocorrelation was calculated with smoothed rate maps.^[^ ^48^, ^64^ ^]^ The autocorrelograms were derived from Pearson's product‐moment correlation coefficient corrected for the edge effects and behavioral occupancy.

### Analysis of Grid Cells

Grid cells were quantified using the gridness score according to previously published methods.^[^ ^37^, ^48^, ^64^ ^]^ First, spatial autocorrelations were calculated using smoothed firing rate maps:

(1) $$r\left(\tau_{x},\tau_{y}\right)=\frac{n\sum \lambda \left(x,y\right)\lambda \left(x-\tau_{x},y-\tau_{y}\right)-\sum \lambda \left(x,y\right)\sum \lambda \left(x-\tau_{x},y-\tau_{y}\right)}{\sqrt{n\sum \lambda {\left(x,y\right)}^{2}-{\left[\sum \lambda \left(x,y\right)\right]}^{2}}\sqrt{n\sum \lambda {\left(x-\tau_{x},y-\tau_{y}\right)}^{2}-{\left[\sum \lambda \left(x-\tau_{x},y-\tau_{y}\right)\right]}^{2}}}$$

where λ(*x*, *y*) is the mean firing rate of the corresponding unit at the coordinate of (*x*, *y*), and the summation was over *n* pixels for both λ(*x*, *y*) and λ(*x* − τ_*x*_,*y* − τ_*y*_) (τ_*x*_ and τ_*y*_ denote the spatial lags). Autocorrelations were not calculated for spatial lags of τ_*x*_, τ_*y*_ where *n* < 20.

Then, the gridness score was computed for each unit by comparing values along a circle centered on the central peak of the auto‐correlogram but excluding the central peak itself, with rotated versions of those values.^[^ ^13^, ^48^ ^]^ Specifically, Pearson's correlations between the circular sample and its rotated versions were calculated, with 60° and 120° angles of rotation in the first group, and 30°, 90°, and 150° angles of rotation in the second group. The gridness score was defined as the minimal difference between any of the coefficients in the first group and any of the coefficients in the second group.

The chance value for defining grid cells was determined by a shuffling process, with the entire sequence of spike trains time‐shifted between 20 s and the whole trail length minus 20 s along the animals trajectory. This shuffling process was repeated 100 times for each cell, generating a total of 128200 permutations for the 1282 MEC units. This shuffling procedure preserved the temporal firing characteristics in the unshuffled data while disrupting the spatial structures at the same time. Cells were defined as grid cells if the gridness score of the recorded cell was larger than both the 99th percentile of the shuffled distribution.

### Analysis of Head‐Direction Cells

The rat's head direction was estimated by the relative position of the two LEDs differentiated through their sizes.^[^ ^9^, ^64^ ^]^ The directional tuning curve for each recorded cell was drawn by plotting the firing rate as a function of the rat's head angle, which was divided into bins of 3° and then smoothed with a 15° mean window filter (2 bins on each side). To minimize the sampling bias, it only included data if all directional bins were covered by the animal before smoothing.^[^ ^64^ ^]^

The strength of directionality was measured by computing the mean vector length (MVL) from the circular distribution of directional firing rates, which was computed using the following equation:

(2) $$\mathrm{MVL}=\frac{\sqrt{{\left(\sum_{1}^{n}F_{i}*\cos\left(\theta_{i}\right)\right)}^{2}+{\left(\sum_{1}^{n}F_{i}*\sin\left(\theta_{i}\right)\right)}^{2}}}{\sum_{1}^{n}F_{i}}$$

where *F_i_* was the firing rate in the bin *i*, θ_*i*_ was the head direction angle in the bin *i*, *n* is the total number of directional bins. The preferred angle of directional tuning was then determined as the head direction angle with the highest firing rate across all directional bins.

HD cell classification was verified using the same method as for grid cells. The distribution of mean vector length was calculated for the entire set of permutation trials from all recorded units, and the 99th percentile threshold derived from the shuffled data was chosen as the threshold. Angular stability was computed by calculating the correlation of firing rates across directional bins generated from the first and second halves of the same trial. The same shuffling procedure was performed to calculate population shuffled distribution of angular stability, and the threshold was determined as the 99th percentile of the shuffled population. Cells were defined as HD cells if the mean vector length and angular stability of the recorded cell were larger than both the 99th percentile of the mean vector length and angular stability in the shuffled distribution.

### Analysis of Bipolar Head‐Direction Cells

To characterize bipolar HD cells, a two‐component circular normal distribution^[^ ^21^ ^]^ was fitted to the directional tuning curve:

(3) $$f=A+Be^{K_{1}cos\left(\theta -\theta_{1}\right)}+Ce^{K_{2}cos\left(\theta -\theta_{2}\right)}$$

Where *f* was the firing rate as a function of head direction θ, θ_1_ and θ_2_ were the primary and secondary preferred directions of the dual peaks, A, B, *K*_1_ and *K*_2_ are all positive parameters. Generally speaking, *K*_1_ and *K*_2_ determine the sharpness of the dual peaks, whereas A was the background firing rate and $Be^{K_{1}}$ and $Ce^{K_{2}}$ were the peak firing rates of the dual peaks. Bipolar HD cells were identified if they met the following criteria: 1) the coefficient of determination (R^2^) exceeding the 99.9th percentile of the shuffled population distribution; 2) the ratio between peak firing rates of the small peak versus the large peak larger than 0.1; 3) the angular stability exceeding the 99th percentile of the shuffled population distribution. For cells that met both criteria of being classified as a unipolar HD cell and a bipolar HD cell, the cell sample was excluded from the unipolar HD sample. By utilizing this standard, 19 out of 106 bipolar HD cells were excluded from the HD population.

### Dimensionality Reduction and Manifold Analysis

Non‐linear dimensionality reduction technique Laplacian Eigenmaps (LEM) was used as previously described.^[^ ^31^, ^65^ ^]^ First, a surrogate trajectory and spike train for each bipolar HD cell was constructed because the number of simultaneously recorded cell was limited. The surrogate spike train of each cell was randomly sampled from the raw spike train of that cell when the head direction in each trajectory was matched. Then, Laplacian Eigenmaps were applied. Briefly, the classification technique k‐Nearest Neighbor (kNN) was used to construct a neighborhood graph to capture the local structure of the data. A simple‐minded method was used that the weight between connected vertices equals 1. Then, the eigenvalues and eigenvectors for the generalized eigenvector problem were computed:

(4) Lf=λDf

where *D* is the diagonal weight matrix with $D_{ii}=\sum_{j}W_{ij}$. *L* = *D* − *W* is the Laplacian matrix. The first eigenvalue was omitted corresponding to eigenvalue 0 and the next 10 eigenvalues were chosen for embedding in the 10D Euclidean space.

### Analysis of Theta‐Rhythmicity

Theta‐rhythmicity was quantified using a previously described method.^[^ ^54^ ^]^ Briefly, to calculate fluctuations of neural activity through the theta cycle, the local field potentials (LFPs) were filtered to extract theta oscillations. For the low‐pass filtering, 4 and 5 Hz were selected as stopband and passband low cut‐off frequencies, respectively, while 10 and 11 Hz were selected as passband and stopband high cut‐off frequencies, respectively. Theta‐rhythmicity was calculated from the fast Fourier transform (FFT)‐based power spectrum of the spike‐train autocorrelation. When the mean spectral power within 1 Hz range of the theta peak within the 4–11 Hz frequency range was at least five times larger than the mean spectral power from 0 Hz to 125 Hz (the ratio defined as theta rhythmic index, TRI), the cell was classified as being theta‐rhythmic. A Hilbert transform was applied to decompose theta oscillation into amplitude and phase, and the instantaneous phase was determined for every spike. The preferred phase was calculated as the mean angle of the resultant vector of the theta phases for all spike times.

### Spike‐Time Cross‐Correlogram

Spike‐time cross‐correlograms between co‐recorded unipolar and bipolar HD cells were derived according to a previous study.^[^ ^66^ ^]^ Briefly, spike‐time cross‐correlograms (0.4 ms binning) between two co‐recorded cells were computed, followed by convolving with a finite window of a partially hollow Gaussian kernel.^[^ ^67^ ^]^ Then, a Poisson distribution was applied to estimate the upper and lower confidence limits. If a short‐latency peak (±4 ms) in the cross‐correlogram was higher than the upper confidence bound, the interactions were considered as putative monosynaptic excitatory connections. For cell pairs co‐recorded on the same tetrode, the 0–1.6 ms range of the cross‐correlogram was not considered as superimposed spikes could not be resolved by the clustering procedure.^[^ ^68^ ^]^ Finally, for two cells recorded on the same tetrode with putative monosynaptic connections, if the cross‐correlogram's −3‐0 ms range was significantly lower than the lower confidence bound, the connection was excluded to account for the possible refractory period from bursting firing.

### Histology

After the final recording session, rats were deeply anaesthetized with sodium pentobarbital (0.01 mL g^−1^) and were perfused intracardially with 1 × phosphate‐buffered saline (PBS) and then with 4% paraformaldehyde (PFA). In one rat (MEC#5), an additional electrolytic lesion was performed before anaesthesia by passing a small current (20 µA, 15 s) through two active electrodes of the microdrive. The brains were removed from the skull and post‐fixed in 4% PFA at 4 °C overnight before transferring into 20 and 30% sucrose/PFA solution sequentially across 72 h. 30 µm thick sagittal sections were serially cut using a cryostat and were mounted on glass slides.

Brain sections were stained with Cresyl Violet acetate (C5042, Sigma–Aldrich, USA), after which tetrode tracks were examined using Olympus Slideview VS200 Digital Slide Scanner (Olympus, Japan). The final positions of the tips of the recording electrodes were estimated from the deepest tetrode track according to the daily notes on tetrode advancement. The tissue shrinkage correction was calculated by dividing the distance between the brain surface and electrode tips by the final depth of the tetrodes. Tetrode traces were confirmed to be located in MEC based on the reference figures (from Figure 179 to 180) published in the seventh edition of *The Rat Brain in Stereotaxic Coordinates*.^[^ ^69^ ^]^

### Statistical Analysis

Statistical analysis was performed using SPSS statistical software (IBM SPSS Statistics 20; USA) and MATLAB (The MathWorks; USA). Mann–Whitney U test and Wilcoxon signed‐rank test were used for unpaired and paired tests between two groups, respectively. Chi‐squared test was used to compare the ratios between two groups. Binomial test was used for determining the chance level. Circular V‐test was used for testing the non‐uniformity of circular data with a specified mean direction. *P* values of < 0.05 were considered significant. n.s., not significant, *P* > 0.05, *P < 0.05, **P < 0.01, and ***P < 0.001. Data are means ± s.e.m.

## Conflict of Interest

The authors declare no conflict of interest.

## Author Contributions

X.L. and X.W. contributed equally to this work. X.L. and S.‐J.Z. performed conceptualization. X.L. and S.‐J.Z. performed surgery. B.D. and X.L. performed data recording. X.L., R.S. and X.W. performed code development. X.L. and X.W. performed data analysis. B.D. and X. L. performed histology. X.L., S.‐Q.L., X.W., and S.‐J.Z. performed result discussion and comments. X.L., S.‐Q.L. and S.‐J.Z. performed writing. L.X., S.‐Q.L., and S.‐J.Z. performed funding acquisition. S.‐J.Z. performed supervision. All authors performed reading and approval.

## Supporting information

Supporting Information

## Data Availability

The data that support the findings of this study are available in the supplementary material of this article.
