# Supplementary material for: Intrinsic Bipolar Head‐Direction Cells in the Medial Entorhinal Cortex
Source: Adv Sci (Weinh). 2024 Aug 29;11(40):2401216. doi: 10.1002/advs.202401216 (PMC11515902; doi:10.1002/advs.202401216)
Supplement: Supplementary file 1 — Supporting Information [file ADVS-11-2401216-s001.docx]

**Supplementary Information**

The supplementary information contains Supplementary **Figures S1** to **S10**.


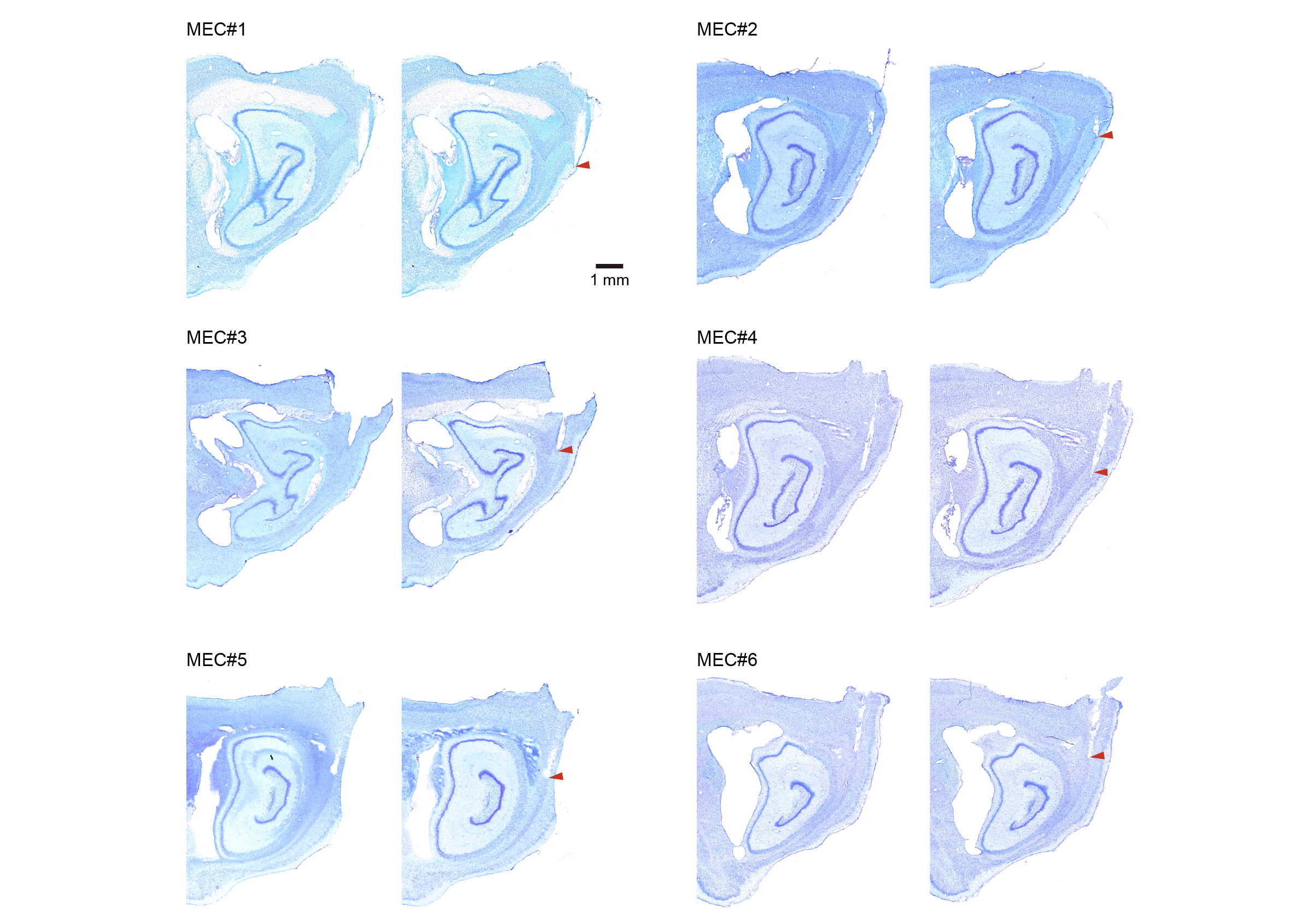


**Supplementary Figure 1. Sagittal Nissl-staining showing tetrode tracks through MEC.**

Nissl-stained sagittal brain sections from six rats showing recording sites in the MEC. Red triangles point to the deepest position of the tetrode. Rat numbers are indicated at the top left. Note that an additional electrolytic lesion was performed for MEC#5. Scale bar, 1 mm.


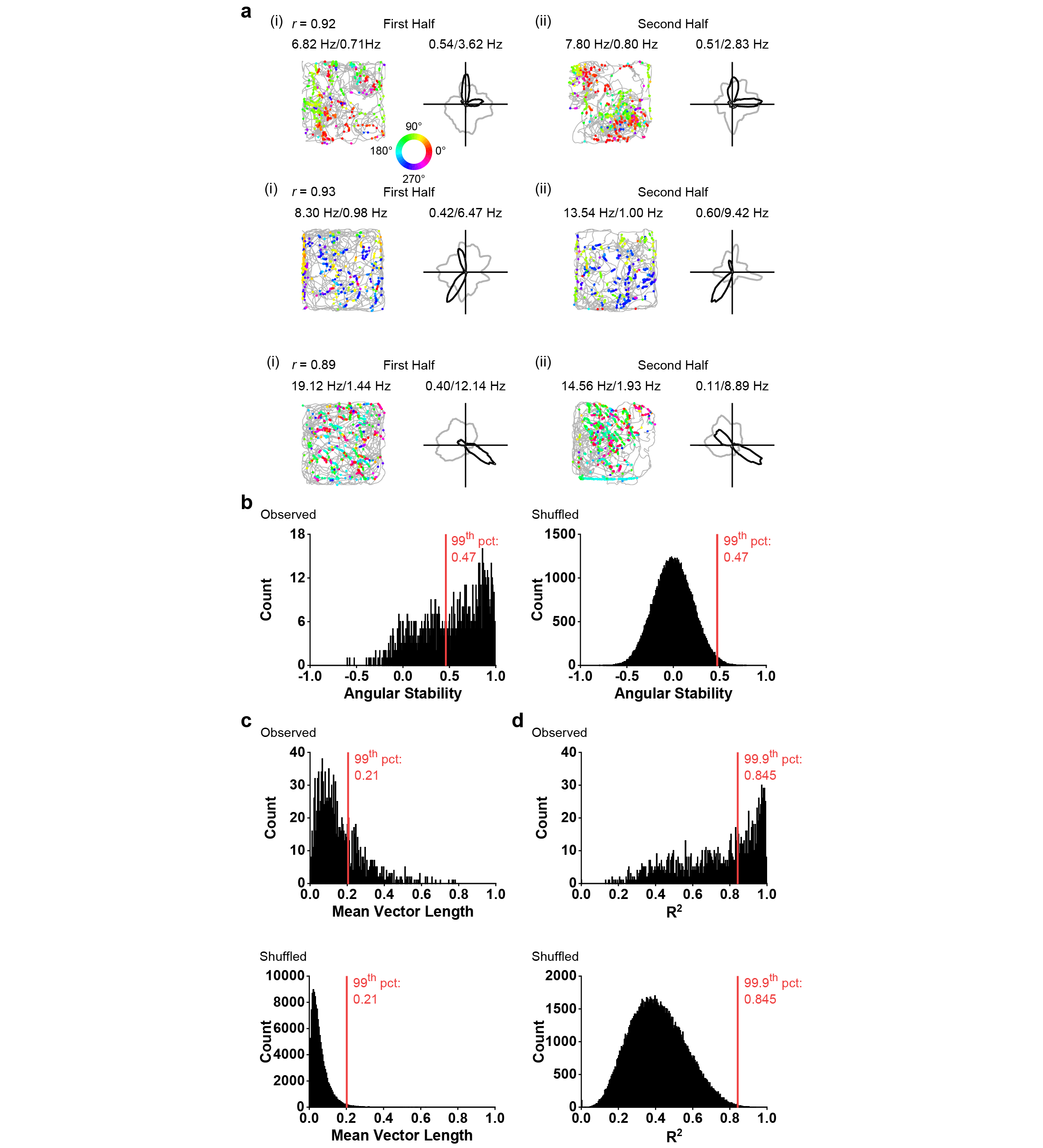


**Supplementary Figure 2. Angular stability of bipolar directional responses.**

(**a**-**c**) Angular stability of the three bipolar HD cells from **Fig. 1a** between the first half and the second half of the same session. Notations and symbols are similar to **Figure. 1**. (**b**) Distribution of angular stability for observed data (left panel) and shuffled data (right panel). Red line indicates the 99^th^ percentile for the angular stability derived from the shuffled data. (**c**) Same as (**b**) except for mean vector length. (**d**) Same as (**b**) except for the 99.9^th^ percentile of R^2^ of two-component circular normal fit.


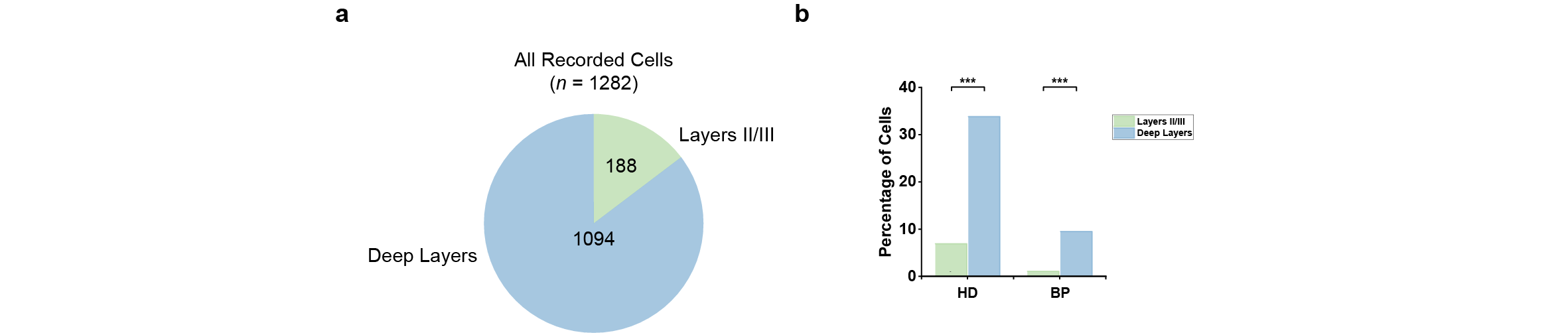


**Supplementary Figure 3. Layer distribution of head-direction cells in MEC.**

(**a**) Proportion of all recorded cells in layers II/III and deep layers IV/V/VI. (**b**) Percentage of unipolar head-direction cells and bipolar head-direction cells recorded from different layers within the MEC in rats.


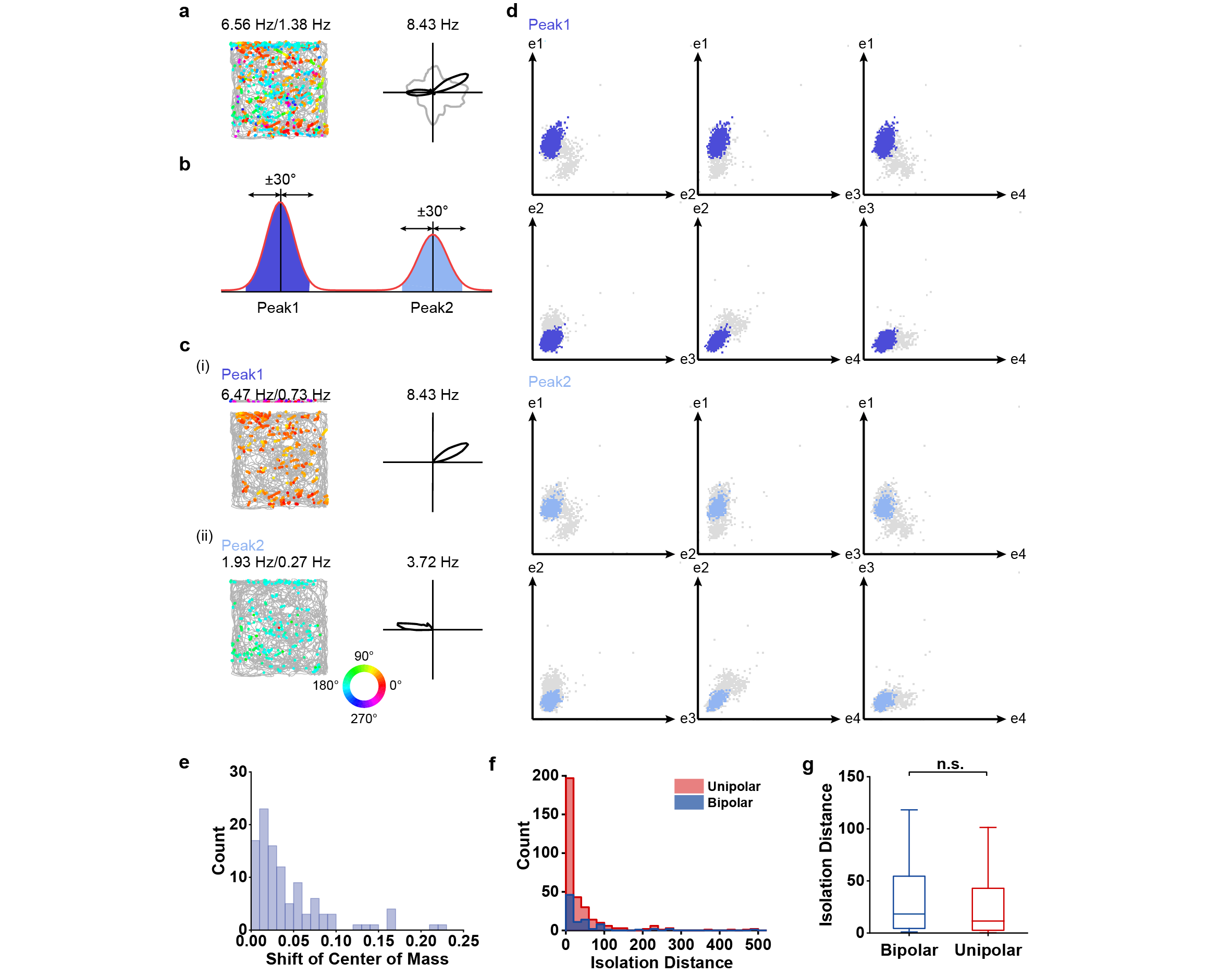


**Supplementary Figure 4. Bipolar head-direction cells are not an artifact of recording from two unipolar head-direction cells.**

(**a**) Separation of spikes from one bipolar HD cells into two clusters based on the preferred directions of the two peaks. First row, one bipolar HD cell. Notations and symbols are similar to **Figure 1**. Second row, a schematic diagram showing the separation of spikes into two clusters based on their preferred directions. When a spike has head directionality within ±30° range of one of the two preferred head directions, it is sorted into the corresponding spike cluster. Third and fourth row, the spatial responses of the two separated spike clusters. (**b**) The separated spike clusters for the bipolar HD cell in (**a**). Scatter plots showing the relationship between peak-to-trough amplitudes for spikes from the first peak (top panel) and the second peak (bottom panel), which are recorded on six combinations of four channels (ch1-ch4) on a specific tetrode from one recording session. Each dot represents a single recorded spike. Dark blue and light blue indicate the cluster from the two separated peaks in (**a**). Grey dots indicate unclustered spikes. (**c**) Histogram showing the cluster separation quantified by the shift of the center of mass between two separated clusters for all bipolar HD cells.


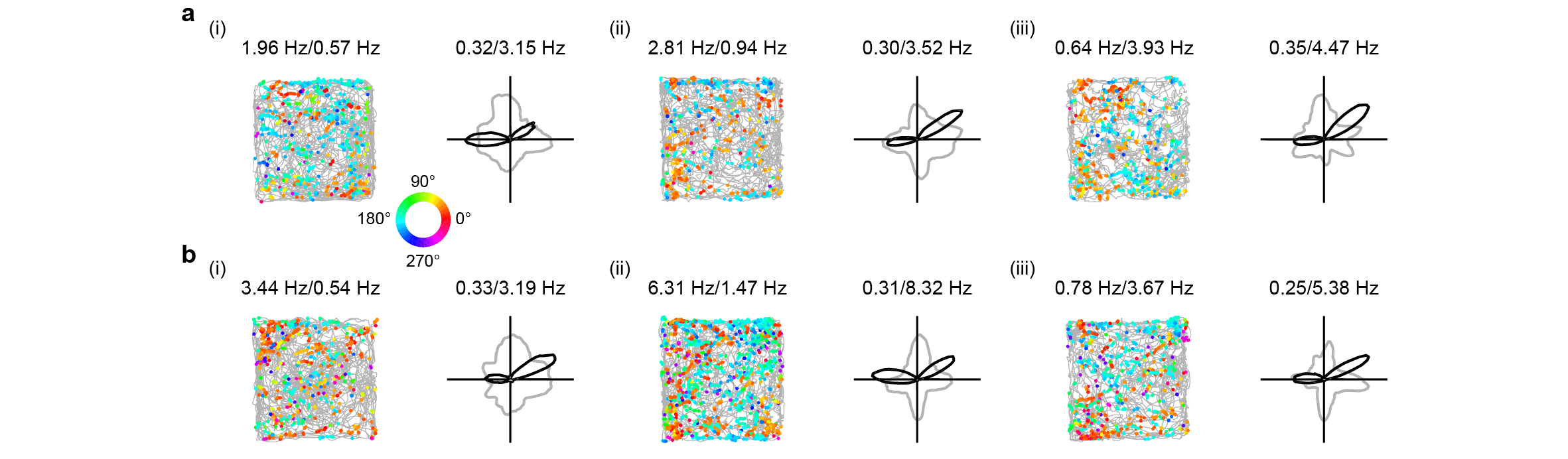


**Supplementary Figure 5. Dynamic change of peak firing rate between the two peaks of bipolar head-direction cell.**

(**a**) Spatial responses of one bipolar HD cell in three consecutive sessions. Notations and symbols are similar to **Figure 1**. (**b**) Spatial responses of the same bipolar HD cell in three consecutive sessions on another day. Note the dynamic change of the peak firing rate of the bipolar HD cell.


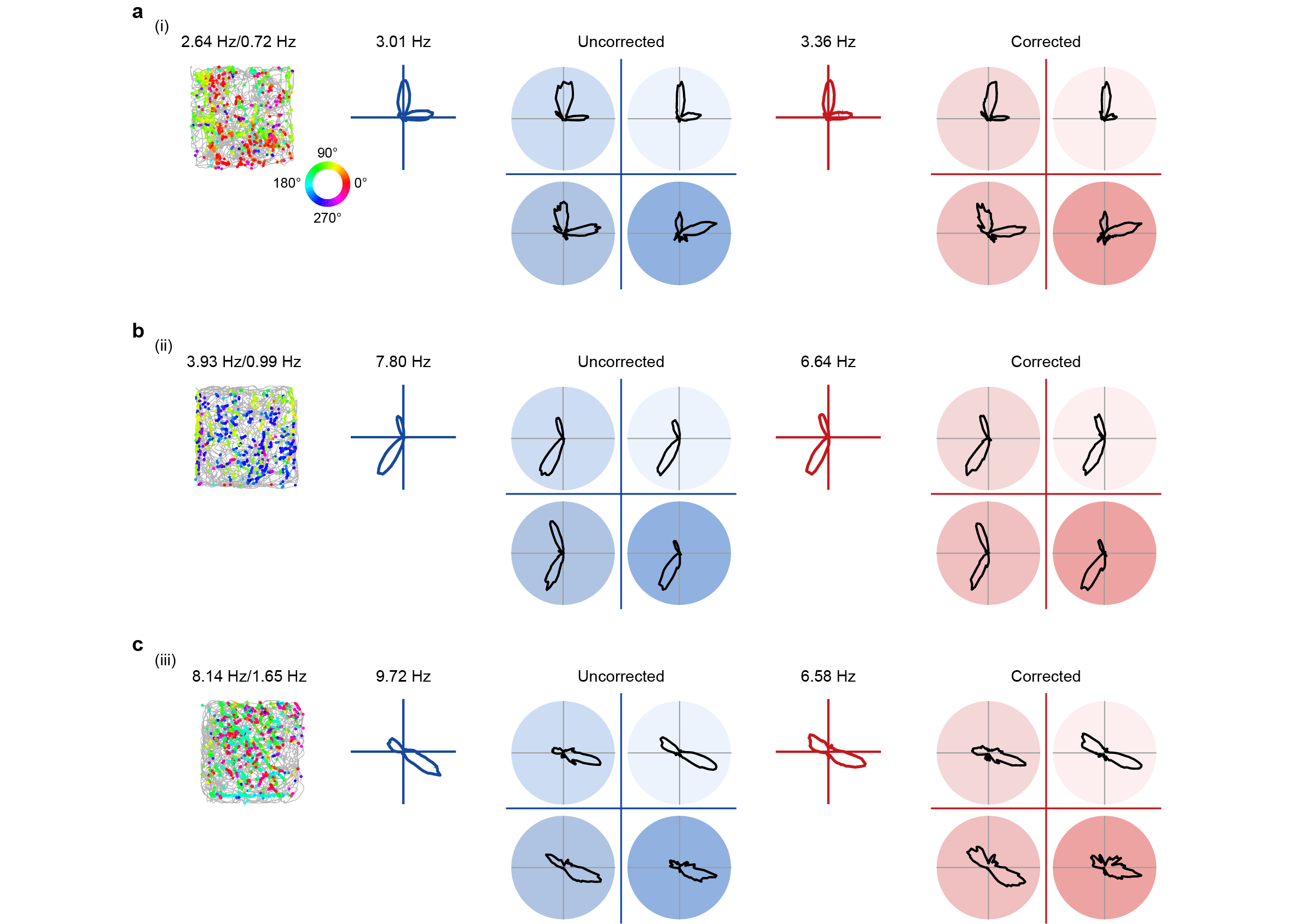


**Supplementary Figure 6. Quantification of head direction selectivity of bipolar head-direction cells using the maximum-likelihood factorial model.**

(**a**-**c**) Comparison of directional tuning of three bipolar HD cells from **Figure 1** using a maximum-likelihood approach. Left panel, color-coded trajectory (grey line) with superimposed directional spike locations (Color circles indicate the corresponding head direction. Color bar shows the directional range 0°-360°). The second and third panels show the uncorrected polar plots for the whole and in each quadrant of the running box. The fourth and fifth panels show the corrected polar plots for the whole and in each quadrant of the running box. Bipolar HD cells preserve the bimodal directional tuning in different parts of the running box regardless of possible inhomogeneous sampling of the animal’s locations and orientations.


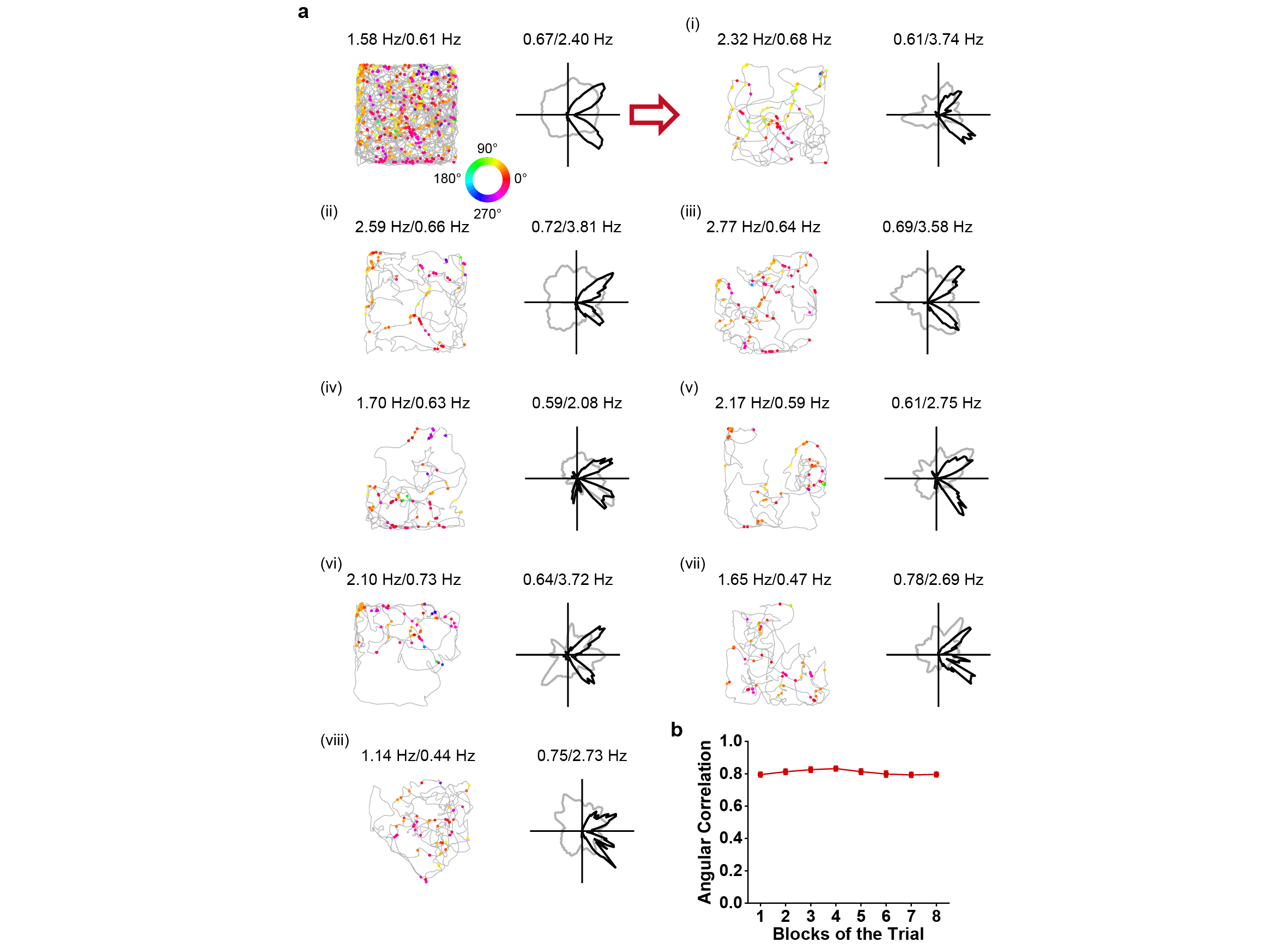


**Supplementary Figure 7. Bipolar directional responses are expressed instantly.**

(**a**) Spatial response of one bipolar HD cell. Notations and symbols are similar to **Figure 1**. The session is broken into eight blocks to show the development of bipolar responses. (**b**) Development of bipolar firing patterns as shown by spatial correlation with the whole session of directional firing rate across eight divided blocks of the same trial (*n* = 106, means ± s.e.m.).


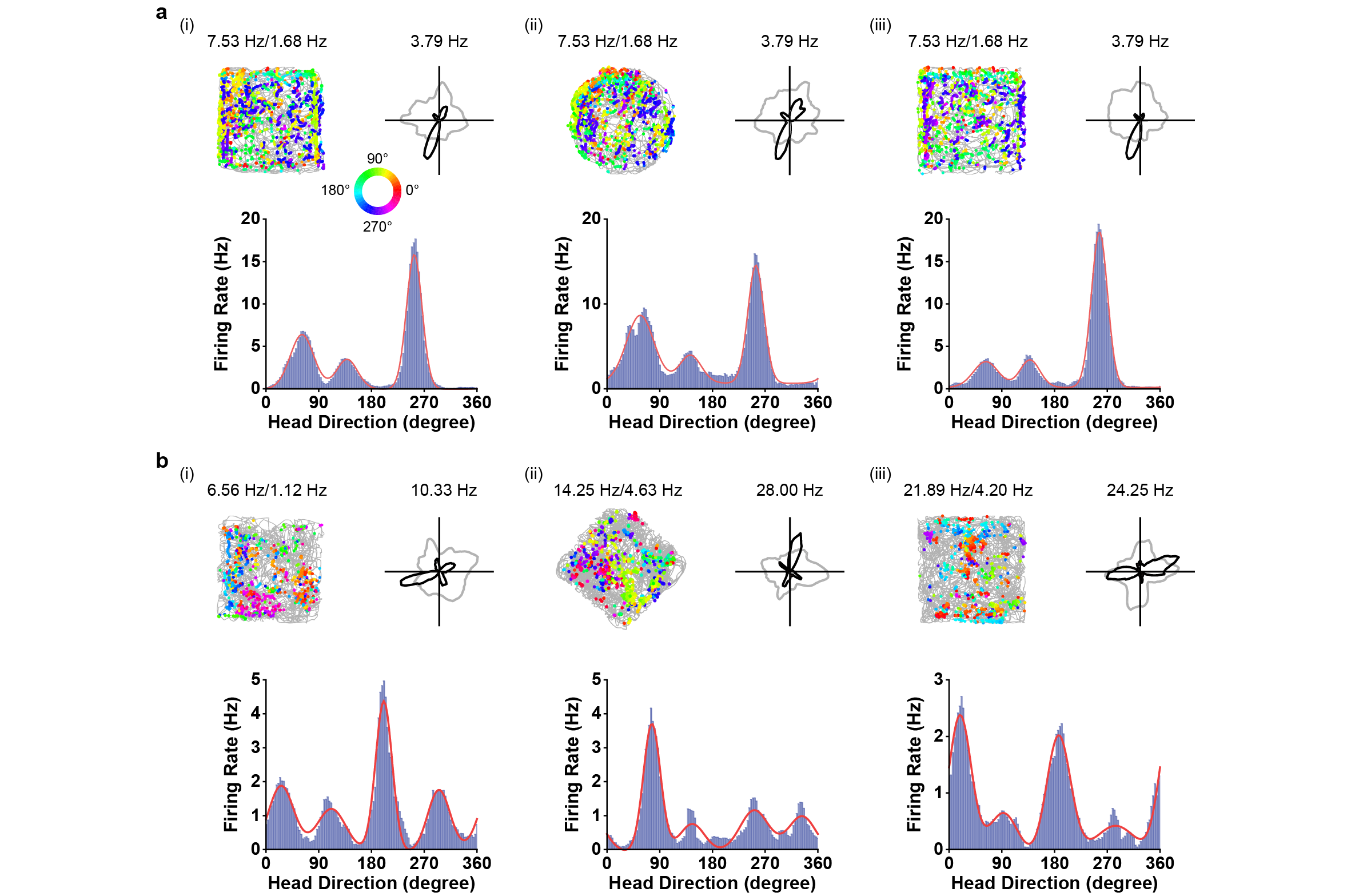


**Supplementary Figure 8. Multipolar head-direction cells in MEC.**

(**a**) One triple-polar HD cell from MEC. From left to right, three consecutive sessions in square, circle and square enclosures. (**b**) One quadruple-polar HD cell in baseline, counterclockwise 45-degree rotation and baseline sessions. Notations and symbols are similar to **Figure 1**.


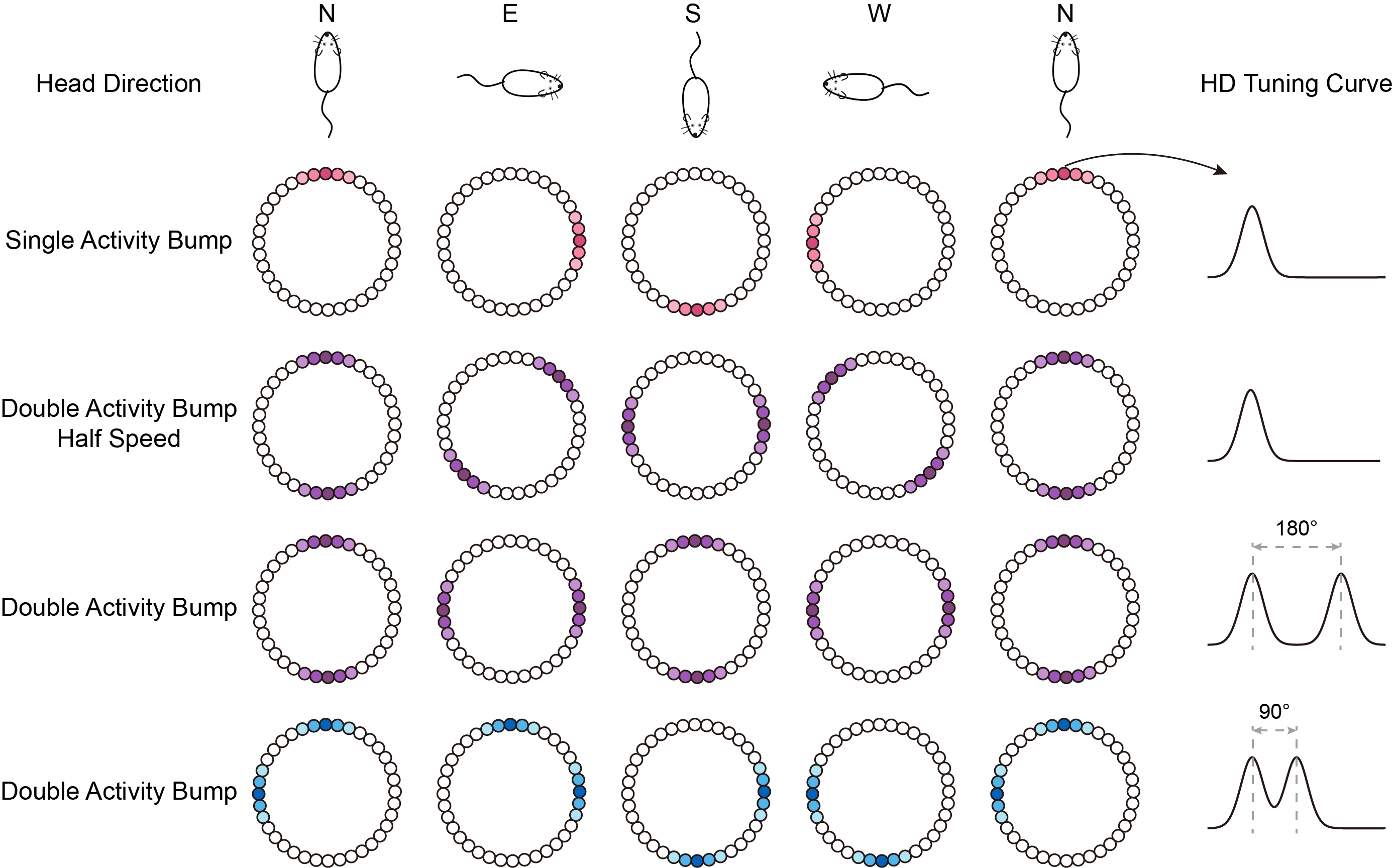


**Supplementary Figure 9. Head-direction ring network.** In the classic ring attractor network model, the head direction (top row) is represented by a single activity bump (second row) in a ring formed of HD cells. The HD tuning curve on the right panel corresponds to the HD cell on the top of the ring, pointed by the arrow. If the ring network has a double activity bump and rotates at half of the speed as the single activity bump, then the tuning curve of the HD cell would remain the same (third row). However, if the ring rotates at the same speed, the tuning curve would exhibit dual peaks, and the angular offset between the two peaks would depend on the separation between the two activity bumps of the ring (Adapted and modified from C. Wang and K. Zhang, *Front Comput Neurosci* 2019, 13, 96).


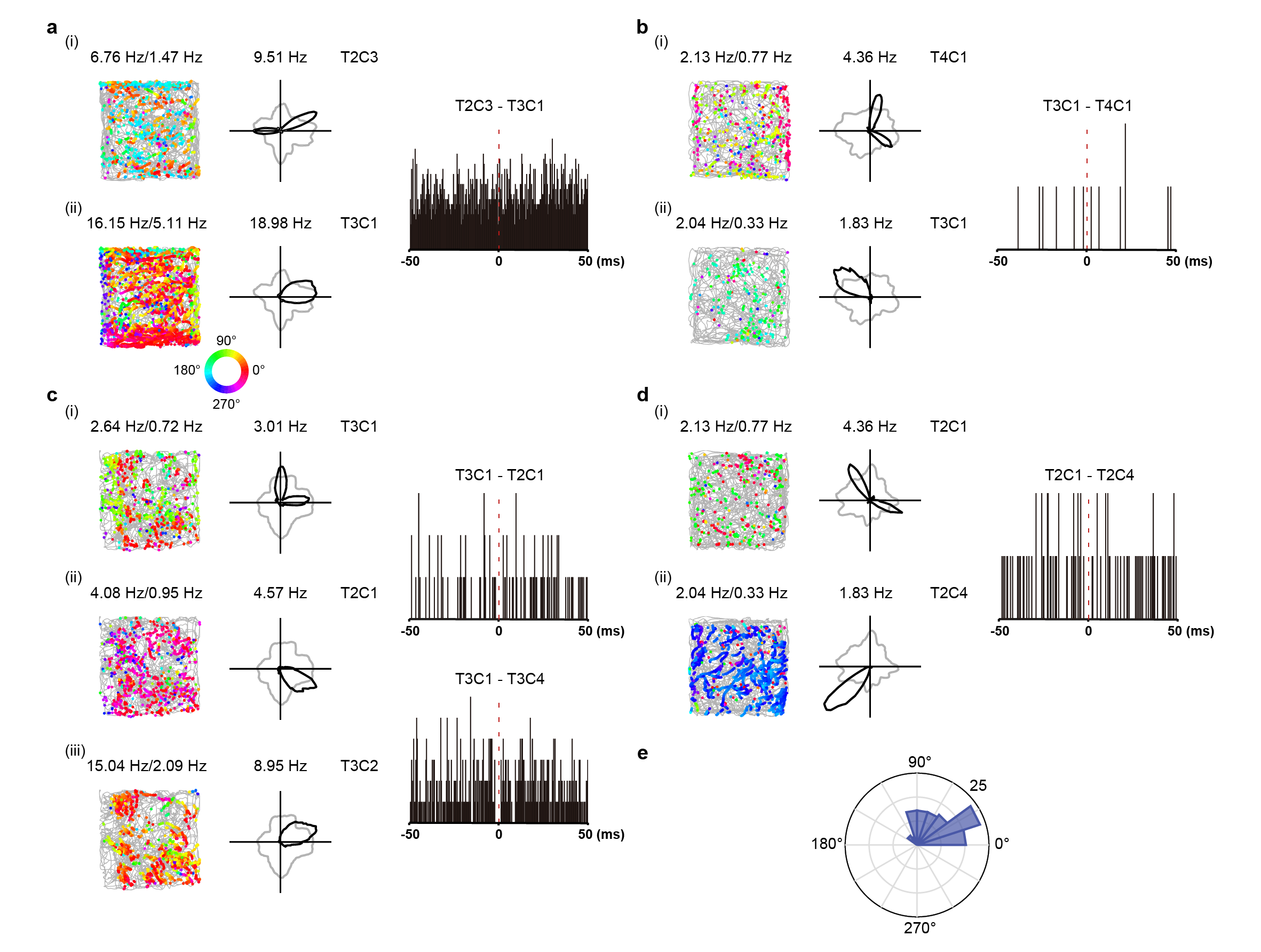


**Supplementary Figure 10. No detectable functional connectivity between MEC unipolar and bipolar HD cells.** (**a**-**d**) Four examples of spike-time cross-correlograms between co-recorded MEC unipolar and bipolar HD cells. Left panel, color-coded trajectory (grey line) with superimposed directional spike locations (Color circles indicate the corresponding head direction. Color bar shows the directional range of 0°-360°. Right panel, HD tuning curves (black) plotted against dwell-time polar plot (grey). Peak firing rate, mean firing rate, and peak angular rate for each cell are labelled at the top of the panels. Corresponding tetrode number and unit number are indicated. Right panels, spike timing cross-correlograms. (**e**) The distribution of minimum angular differences between preferred firing direction of unipolar HD cells and two preferred firing directions of bipolar HD cells.
